# Functional Near-Infrared Spectroscopy to Study Cerebral Hemodynamics in Older Adults During Cognitive and Motor Tasks: A Review

**DOI:** 10.3389/fnagi.2019.00367

**Published:** 2020-01-21

**Authors:** Cristina Udina, Stella Avtzi, Turgut Durduran, Roee Holtzer, Andrea L. Rosso, Carmina Castellano-Tejedor, Laura-Monica Perez, Luis Soto-Bagaria, Marco Inzitari

**Affiliations:** ^1^Parc Sanitari Pere Virgili, Barcelona, Spain; ^2^RE-FiT Barcelona Research Group, Vall d’Hebrón Institute of Research, Barcelona, Spain; ^3^Department of Medicine, Universitat Autònoma de Barcelona, Barcelona, Spain; ^4^Institut de Ciències Fotòniques, The Barcelona Institute of Science and Technology, Barcelona, Spain; ^5^Institució Catalana de Recerca i Estudis Avançats, Barcelona, Spain; ^6^Ferkauf Graduate School of Psychology, Yeshiva University, New York, NY, United States; ^7^Department of Neurology, Albert Einstein College of Medicine, New York, NY, United States; ^8^Department of Epidemiology, Graduate School of Public Health, University of Pittsburgh, Pittsburgh, PA, United States

**Keywords:** functional Near-Infrared Spectroscopy, gait, dual task, motor task, cognition, older adults, prefrontal cortex, cerebral hemodynamics

## Abstract

The integrity of the frontal areas of the brain, specifically the prefrontal cortex, are critical to preserve cognition and mobility in late life. Prefrontal cortex regions are involved in executive functions and gait control and have been related to the performance of dual-tasks. Dual-task performance assessment may help identify older adults at risk of negative health outcomes. As an alternative to neuroimaging techniques that do not allow assessment during actual motion, functional Near-Infrared Spectroscopy (fNIRS) is a non-invasive technique that can assess neural activation through the measurement of cortical oxygenated and deoxygenated hemoglobin levels, while the person is performing a motor task in a natural environment as well as during cognitive tasks. The aim of this review was to describe the use of fNIRS to study frontal lobe hemodynamics during cognitive, motor and dual-tasks in older adults. From the 46 included publications, 20 studies used only cognitive tasks, three studies used motor tasks and 23 used dual-tasks. Our findings suggest that fNIRS detects changes in frontal activation in older adults (cognitively healthy and mild cognitive impairment), especially while performing cognitive and dual-tasks. In both the comparison between older and younger adults, and in people with different neurological conditions, compared to healthier controls, the prefrontal cortex seems to experience a higher activation, which could be interpreted in the context of proposed neural inefficiency and limited capacity models. Further research is needed to establish standardized fNIRS protocols, study the cerebral hemodynamic in different neurological and systemic conditions that might influence cortical activation and explore its role in predicting incident health outcomes such as dementia.

## Introduction

The worldwide aging of the population makes tackling aging-associated disability an urgent priority. Cognitive impairment and mobility disability are key contributors to dementia and loss of independence in the activities of daily living and have a synergistic effect (Verghese et al., 2014). The integrity of the frontal areas of the brain, specifically the PFC, are critical to preserve cognition and mobility in late life (Beauchet et al., 2016). PFC regions carry out executive functions, i.e., higher order cognitive functions essential to plan and execute complex goal-directed actions, which are also key for motor control in older adults (Inzitari et al., 2007). The loss of integrity in frontal or prefrontal regions, either due to neurodegeneration, cerebrovascular disease or due to their interactions, contributes to the development of dementia (Burgmans et al., 2009; Kisler et al., 2017) and mobility impairments (De Laat et al., 2011). The PFC has also been implicated in performance of DT (Sala et al., 1995; Dux et al., 2006; Filmer et al., 2013), that are motor tasks performed simultaneously with a secondary, usually a cognitive task. DT increases the cognitive demand of walking and potentially results in a decrease in task performance in one or both tasks relative to when the tasks are performed separately as ST. DT performance assessment may help identify older adults at higher risk of incident cognitive decline (Ceïde et al., 2018; Rosso et al., 2019), disability, frailty and mortality (Verghese et al., 2012). One of the goals of the study of cognitive aging is to elucidate neural mechanisms that underlie the ability of the aging brain to cope with decline in cognitive functions and efficiency. Several hypothesis have been described and there is still no consensus regarding definitions of several concepts (Cabeza et al., 2018). Two of the previously described hyphotheses are: the “neural inefficiency hypothesis” (Rypma and D’Esposito, 2000; Holtzer et al., 2009) or “compensation by upregulation” (Cabeza et al., 2018), according to which older adults show increased activity of the same networks recruited by younger counterparts in order to meet behavioral demands, and the “capacity limitation hypothesis” (Cabeza, 2004; Holtzer et al., 2009) which postulates that older adults, while recruiting the same brain networks as young adults, would show a reduced activation compared to their younger counterparts (Holtzer et al., 2009; Stern, 2009).

Classic clinical and epidemiological studies have based their assessment of PFC on a static, structural basis, mainly through magnetic resonance imaging (MRI) techniques, which have shown a contribution of both cortical frontal and PFC volumes (Rosano et al., 2008; Weinstein et al., 2012) and subcortical alterations to executive dysfunction/dementia (Jokinen et al., 2009) and mobility limitations (Baezner et al., 2008). In addition, functional neuroimaging techniques, such as functional MRI (fMRI), allow the study of PFC by assessing the hemodynamic changes due to neurovascular coupling that are triggered by its neural activation (Buchbinder, 2016). fMRI studies assess whole brain function with a relatively high spatial resolution, are non-invasive and the most used technique to date to assess neural activity during specific task activation (Rosen and Savoy, 2012). Several fMRI studies have demonstrated the relevance of PFC for executive functions (Wager et al., 2004; Venkatraman et al., 2010; Yaple et al., 2019) and DT (Szameitat et al., 2002; Dux et al., 2006; Jurado and Rosselli, 2007). Limitations of both MRI and fMRI include their relatively high cost, unsuitability for many older adults due to metal implants in the body, claustrophobia or inability to lie still for long periods. Further, due to the nature of the scanner, the tasks are carried out in unnatural environments which may alter their relevance to the real-world and do not allow functional analysis of brain activity during locomotion. Imagined gait has been used as a way to study the neural correlates of locomotion with fMRI (Zwergal et al., 2012; Blumen et al., 2014); however, it is not entirely clear how well this mimics brain activation during actual walking. Other options, although they do not allow online assessment of gait either, include PET studies after walk trials with administration of fludeoxyglucose-18 tracer (la Fougère et al., 2010). We refer the reader to Holtzer et al. (2014) for a comprehensive review on neuroimaging of locomotion in aging.

Emerging alternatives to fMRI, based on near-infrared diffuse optical techniques, allow measurements in more realistic environments and during motion (Boas et al., 2014; Scholkmann et al., 2014). Accumulating evidence supports the use of these techniques for the study of frontal hemodynamic and metabolic changes (Agbangla et al., 2017; Gramigna et al., 2017). These diffuse optical techniques such as fNIRS (Durduran et al., 2010; Ferrari and Quaresima, 2012) allow the study of tissue composition by emitting near-infrared light (∼650–950 nm) into biological tissue and collecting the photons that undergo multiple scattering and absorption (i.e., diffuse) and emerge few centimeters away from the injection point (Delpy and Cope, 1997; Durduran et al., 2010). At these wavelengths the main absorbers in tissues, i.e., O_2_Hb and HHb, differentially absorb light in a wavelength dependent manner. Therefore, most common fNIRS methods can relate changes in the detected light intensity at different wavelengths to changes in oxygenated and deoxygenated hemoglobin concentrations by utilizing the modified Beer-Lambert law (Scholkmann et al., 2014). This is a signal similar to the blood oxygen level dependent (BOLD) signal from fMRI but can be obtained by portable (even wearable) instrumentation and flexible fiber-optic probes. The majority of the systems are using source and detector probes placed on the scalp of the head. The most common source-detector separations are of few centimeters. Able to detect signal coming from superficial cortical layers (Ferrari and Quaresima, 2012), fNIRS measurement is based on the neurovascular coupling (oxygen consumption to meet energy demands in activated cerebral areas cause an increase in blood flow resulting in an increase of O_2_Hb and decrease of HHb) and both the analysis and acquisition methods are still being developed with O_2_Hb changes appearing more reliable as a marker of brain activation since it has shown high reproducibility and stability over time (Plichta et al., 2006) and has the highest correlation to fMRI BOLD measures (Strangman et al., 2002). fNIRS studies usually consist of a combination of resting periods, to assess baseline brain activity, and different kinds of tasks. Brain activation is then calculated by comparing hemoglobin measurements at baseline and during the task, although there is a high heterogeneity in data processing and analysis methods. Regarding its advantages, fNIRS is a lower cost modality than fMRI, usable at point-of-care, and allows measurements during mobility tasks. These advantatges allow the potential use of the technique to assess cerebral blood flow and oxygenation with application in different pathologies (e.g., stroke, psychiatric disorders,…) resulting thus in continuous growth on relevant literature (Noda et al., 2017; Giacalone et al., 2019). However, the main limitations of most fNIRS devices include: (i) the limited penetration depth, allowing only the interrogation of superficial layers of the cortex in the adult brain, (ii) the assessment of a limited portion of the cortical surface with often a low spatial resolution with the probes that are attached to the scalp, not allowing complete whole-brain imaging, (iii) issues with extracerebral contamination from superficial tissues (i.e., cutaneous or skull perfusion) and (iv) motion artifacts.

Recent studies have expanded the use of fNIRS in the assessment of PFC of older adults during cognitive or motor tests (Verghese et al., 2017). These studies show changes in PFC hemodynamics during the execution of cognitive or motor tasks, and also report differences according to the person’s age and cognitive function. However, these findings are still preliminary and it is not yet clear if there is a specific pattern according to age or cognitive status, nor about how these differences should be interpreted. Recently published reviews have assessed the results of studies on fNIRS during cognitive tasks (Herold et al., 2018) or dual tasks (Gramigna et al., 2017; Herold et al., 2017; Leone et al., 2017; Vitorio et al., 2017; Stuart et al., 2018; Kahya et al., 2019) and some of them have chosen to focus on specific clinical profiles (Gramigna et al., 2017; Vitorio et al., 2017) or on methodological aspects such as fNIRS signal processing (Herold et al., 2017, 2018; Vitorio et al., 2017). To the best of our knowledge, our review is the first to focus specifically on older adults regardless of their clinical profile and to assess, from a clinical point of view, studies using only cognitive or motor tasks, as well as DTs.

The aim of this review is to describe, through an updated literature search, the use of optical techniques, specifically fNIRS, to study brain hemodynamics, with a focus on frontal regions, in relation to cognitive and physical function in normal and pathological older adult populations.

## Methods

This is a narrative review. We performed, however, a search using pre-set criteria, to make sure that we considered all the relevant articles on the topic. We included manuscripts that have aimed to study frontal and prefrontal lobe hemodynamics (excluding those focusing on other brain regions) using fNIRS to measure oxygenated and deoxygenated hemoglobin levels during cognitive, motor and DTs in older adults. Articles were included if the mean age of the sample or a separately analyzed subgroup was 60 years or older. Review articles, studies assessing change in cerebral hemodynamics after an intervention, those not written in English and those that do not describe the age of the participants in the manuscript were excluded. In order to focus on most recent literature, we limited the publication date to the previous 5 years. The last search was performed on August 29th, 2018.

The article selection was performed in three phases (review of titles, abstracts, and full-texts). Two independent reviewers (CU and MI) reviewed the titles and abstracts resulting from the search, in order to assess potential inclusion. From the selected articles, we performed a full manuscript review to assess if the article met the eligibility criteria. Discrepancies were solved through consensus.

## Results

### General Description

**FIGURE 1:**
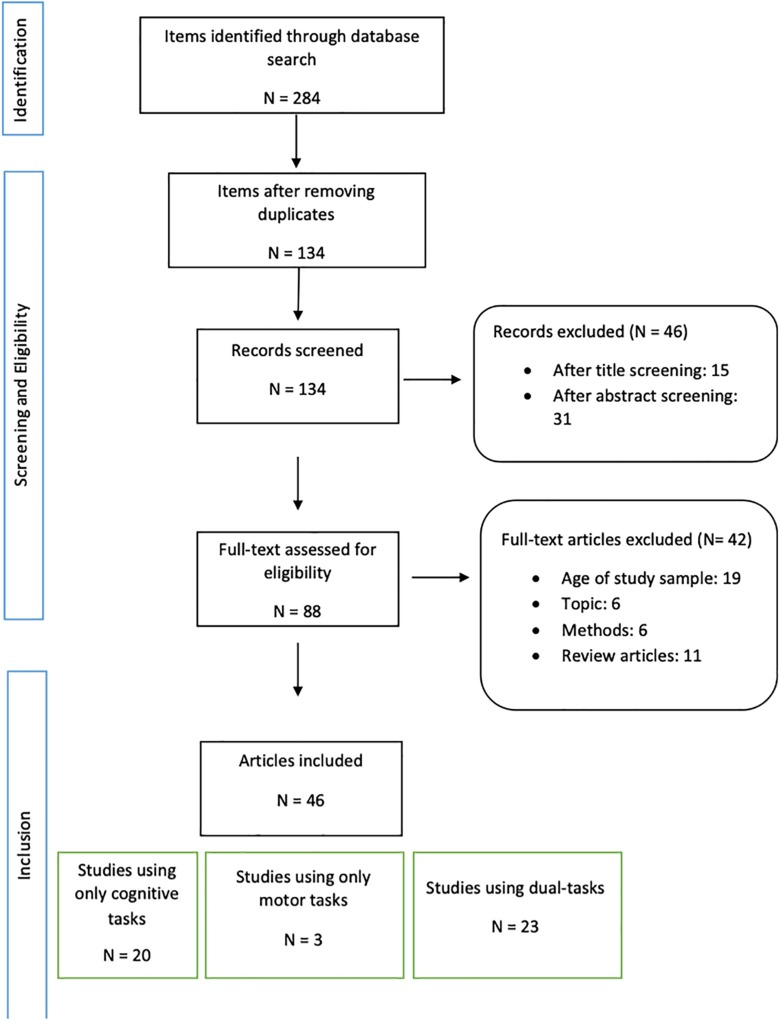
Flow chart diagram of the review process.

As depicted in the flow-chart (Figure 1), after removing duplicates, our search resulted in 134 items. After excluding records by title and abstract screening (*n* = 46), 89 full-text articles were assessed for eligibility. Studies not meeting the above described eligibility criteria such as sample/subgroup mean age (*n* = 19), the aim/topic focus of our review (*n* = 6) (i.e., use of NIRS to monitor cancer treatment), methodological aspects of the design of studies (*n* = 6) (i.e., different location of the probes or NIRS measures performed to assess the effect of an intervention) and review articles (*n* = 11), were excluded. We finally included 46 articles in our review.

Of the 46 included articles, 13 included a mix of younger and older participants (Heilbronner and Münte, 2013; Ohsugi et al., 2013; Beurskens et al., 2014; Müller et al., 2014; Oboshi et al., 2014; Hernandez et al., 2016; Bierre et al., 2017; Mirelman et al., 2017; Rosso et al., 2017; Hawkins et al., 2018) whereas 29 included only older adults (Doi et al., 2013; Heinzel et al., 2013, 2015; Niu et al., 2013; Clark et al., 2014; Vermeij et al., 2014; Dupuy et al., 2015; Holtzer et al., 2015, 2016, 2017a,b, 2018a,b; Laguë-Beauvais et al., 2015; Al-Yahya et al., 2016; Maidan et al., 2016, 2017; Mahoney et al., 2016; Nieuwhof et al., 2016; Osofundiya et al., 2016; Takeuchi et al., 2016; Uemura et al., 2016; Yeung et al., 2016a, b; Chen et al., 2017; Huppert et al., 2017; Verghese et al., 2017; Yap et al., 2017; Halliday et al., 2018; Katzorke et al., 2018; Lucas et al., 2018; Mori et al., 2018; Thumm et al., 2018). Moreover, 26 studies included only cognitively normal participants (Heilbronner and Münte, 2013; Heinzel et al., 2013, 2015; Ohsugi et al., 2013; Beurskens et al., 2014; Clark et al., 2014; Müller et al., 2014; Oboshi et al., 2014; Vermeij et al., 2014; Holtzer et al., 2015, 2016, 2017a,b, 2018a,b; Osofundiya et al., 2016; Bierre et al., 2017; Chen et al., 2017; Huppert et al., 2017; Mirelman et al., 2017; Rosso et al., 2017; Verghese et al., 2017; Halliday et al., 2018; Lucas et al., 2018), seven compared participants with different cognitive status [without cognitive impairment, with MCI or with mild AD] (Doi et al., 2013; Niu et al., 2013; Al-Yahya et al., 2016; Uemura et al., 2016; Yeung et al., 2016a, b; Yap et al., 2017; Katzorke et al., 2018), three studies focused on older adults with previous history of stroke (Al-Yahya et al., 2016; Hawkins et al., 2018; Mori et al., 2018), five assessed patients with parkinsonian syndromes (Mahoney et al., 2016; Maidan et al., 2016, 2017; Nieuwhof et al., 2016; Thumm et al., 2018) and two with Multiple Sclerosis (Hernandez et al., 2016; Chaparro et al., 2017).

Looking at the older adults subgroups that were included in the studies, there was a wide range of mean ages, from 61 ± 4 (Hernandez et al., 2016) to 88.1 ± 6 (Huppert et al., 2017). The largest sample size was 1052 participants (Heinzel et al., 2015) while a sample of 12 older adults was the smallest (Nieuwhof et al., 2016). Most source populations were community-dwelling but two studies included older adults living in nursing home (Osofundiya et al., 2016; Huppert et al., 2017). Ten studies did not describe the participant setting (Niu et al., 2013; Oboshi et al., 2014; Al-Yahya et al., 2016; Uemura et al., 2016; Maidan et al., 2017; Mirelman et al., 2017; Rosso et al., 2017; Katzorke et al., 2018; Mori et al., 2018; Thumm et al., 2018).

The majority, 29 studies, used O_2_Hb to assess brain activation while nine studies (Heilbronner and Münte, 2013; Beurskens et al., 2014; Müller et al., 2014; Al-Yahya et al., 2016; Hyodo et al., 2016; Nieuwhof et al., 2016; Rosso et al., 2017; Halliday et al., 2018; Katzorke et al., 2018) used both O_2_Hb and HHb and one used only Total Hb (Huppert et al., 2017). Two studies calculated the Total Oxygenation Index (O_2_Hb/Total Hb x 100) in order to assess brain hemodynamics (Clark et al., 2014; Bierre et al., 2017). In the following paragraphs, we will use the term activation to refer to changes in these hemoglobin indices.

Twenty-three studies measuring single cognitive or motor tasks performed intra-group comparisons of the cerebral activation during different tasks and the rest periods (see articles listed in Tables 1, 2A), whereas the other 23 studies compared cerebral hemodynamics between single and DT (see articles listed in Table 2B). Twenty-four studies performed comparisons of cerebral activation patterns between different groups (either young vs. old, MCI vs. cognitively normal, healthy vs. stroke etc.) (Heilbronner and Münte, 2013; Niu et al., 2013; Ohsugi et al., 2013; Beurskens et al., 2014; Müller et al., 2014; Oboshi et al., 2014; Laguë-Beauvais et al., 2015; Al-Yahya et al., 2016; Hernandez et al., 2016; Maidan et al., 2016, 2017; Mahoney et al., 2016; Osofundiya et al., 2016; Takeuchi et al., 2016; Uemura et al., 2016; Yeung et al., 2016a, b; Bierre et al., 2017; Mirelman et al., 2017; Rosso et al., 2017; Yap et al., 2017; Hawkins et al., 2018; Katzorke et al., 2018; Mori et al., 2018).

**TABLE 1:** Summary of the studies assessing fNIRS measures during cognitive tasks in older adults.

First author (Journal, year), Country	Sample size (N) Clinical characteristics of the sample (mean age +SD) NIRS optodes localization	Paradigm description	Main fNIRS results
**Verbal fluency**			
**Heinzel** (Neurobiol Aging, 2013), Germany (Heinzel et al., 2013)	*N* = 325 Healthy (64.6 ± 7.3). Prefrontal, temporal and parietal.	Tasks: – Phonetic verbal fluency. –Semantic verbal fluency. –Control task: reciting week days. Three trials: 30 s each. Rest: 30 sec after each trial.	With increasing age: ⇓: Lower activation on bilateral inferior frontal junction during verbal fluency. Increased bilateral activation at middle frontal gyri and supramarginal gyri.
**Heinzel** (PLoS One 2015), Germany (Heinzel et al., 2015)	*N* = 1052 Healthy (65.2 ± 6.8). Prefrontal, parietal and fronto-temporal.	Tasks: –Phonetic verbal fluency. –Semantic verbal fluency. –Control task: reciting week days. Three trials: 30 s each. Rest: 30 sec after each trial.	⇑: Increased activation during both verbal fluencies (compared to control task). Stronger response in phonological than semantic (increased activation in right prefrontal and bilateral inferior parietal regions extending toward postcentral gyri and decreased in bilateral fronto-temporal areas).
**Yeung** (Front Aging Neurosci., 2016), China (Yeung et al., 2016a)	*N* = 52 MCI (69.1 ± 6.2); Healthy (68.8 ± 6.1). Prefrontal.	Tasks: – Semantic verbal fluency. Two task blocks, 60 s each. Rest: repeat “1, 2, 3, 4” out loud. Before and after VF task.	⇑: Increased O_2_Hb bilaterally during verbal fluency in both groups. No significant group differences. Control group showed left lateralization of frontal lobe activation (whereas MCI group did not).
**Yap** (Front Aging Neurosci., 2017), Malaysia (Yap et al., 2017)	*N* = 61 Healthy (72.6 ± 8.5); MCI (73.1 ± 8.2); Mild AD (74.7 ± 10). Prefrontal and part of temporal.	Task: – Semantic verbal fluency (60 s). Rest: 20 s before and after task.	⇑: Highest O_2_Hb increase during task was observed in MCI followed by healthy and mild AD.
**Katzorke** (Psychiatry Res Neuroimaging, 2018), Germany (Katzorke et al., 2018)	*N* = 110 Healthy (74.2 ± 1.6); MCI (74.0 ± 1.6). Fronto-temporal.	Tasks: – Letter verbal fluency. – Semantic verbal fluency. – Control (weekdays). Three trials per task: 30 sec each. Rest: 30 s after each task.	⇓: Decreased PFC activation during semantic verbal fluency in MCI compared to healthy controls (but not during phonological verbal fluency).
**N-back tasks**			
**Niu** (CNS Neurosci Ther., 2013), China (Niu et al., 2013)	*N* = 24 MCI (64.8 ± 7.2); healthy (63.5 ± 5.3). Frontal, parietal and temporal.	Task: –Digit N-back task (0-back and 1-back conditions). Three blocks in each condition: 20 trials for each block followed by 1000 ms interstimulus period. Rest: No rest time specified.	⇓: MCI participants showed lower O_2_Hb concentrations in the left dorsolateral PFC, right supplementary motor area and left superior temporal regions compared to control group.
**Vermeij** (Front Aging Neurosci., 2014), Netherlands (Vermeij et al., 2014)	*N* = 18 Healthy older adults (70.8 ± 5.0). Prefrontal.	Task: –Spatial N-back (0-back, 1-back and 2-back conditions). 60 trials (500 ms each) with 3000 ms interval between trials. Rest: Initial 1 min-baseline (staring at screen).	⇑: Increased working-memory load associated increased prefrontal activation and decreased performance.
**Yeung** (Dement Geriatr Cogn Disord., 2016), China (Yeung et al., 2016b)	*N* = 52 MCI (69.1 ± 6.3); cognitively normal (68.8 ± 6.1). Frontal.	Task: –Digit N-back task (0-back and 2-back conditions). 20 trials (1000 ms each) followed by 1000 ms interval between trials. Rest: 30 s between blocks.	⇓: MCI group did not show frontal activation. Tended to reduce activation with high working memory load. ⇑: Control group: frontal activation in high working memory load (2-back condition).
**Other tests for executive functions**			
**Heilbronner** (Neuroimage, 2013), Germany (Heilbronner and Münte, 2013)	*N* = 35. Healthy older adults (68 ± 1.4); younger adults group (23.1 ± 0.4). Frontotemporal.	Task: –Cognitive Go/No Go inhibition task: Go stimulus: press button; No Go stimulus: inhibit pressing button. 1083 stimuli in 5 trials. Rest: Rest in a self-paced manner.	⇑: Older adults showed activation in frontal areas. Compared to young participants, activation shifted rostrally (left hemisphere) and dorsally (right hemisphere) in older adults.
**^∗^ Albinet** (Front Aging Neurosci., 2014), France (Albinet et al., 2014)	*N* = 40 Healthy old adults: high-fit (67.32 ± 4.48); low-fit (68.88 ± 3.87) Prefrontal	Task: –Random Number Generation: participants asked to say random number when heard a tone. Fast pace (tone/1 s) and slow pace (tone/1.5 s). Two trials of 100 responses at each pace. –Control: count in order from one to nine.	⇑: Increasing activation in relation to task difficulty. High-fit group showed greater increase in O_2_Hb.
**Müller** (Neuropsychologia, 2014), Germany (Müller et al., 2014)	*N* = 40 Older adults (70.9 ± 3.5); younger adults (25.7 ± 3.0). PFC, motor and premotor regions.	Task: –Adapted version of Trail Making Test (TMT) A and B. –Control task: retrace 90 interconnected circles. Each test presented three times (30 s each). Rest: 30 s after each test.	⇑: Older adults showed bilateral ventrolateral and dorsolateral prefrontal and premotor cortex activation during TMT-B (more channels active in the right hemisphere). Additional activation in medial and lateral PFC in elderly (younger participants show more ventral PFC, especially in the left hemisphere).
**Oboshi** (PLoS One, 2014), Japan (Oboshi et al., 2014)	*N* = 120 Healthy older adults (71.0 ± 6.4); younger adults (21.7 ± 3.3). Prefrontal.	Task: –Visual working memory task. Six blocks (28.8 s each). Rest: 30 s.	⇑: Older adults: O_2_Hb increase during working memory task. Young adults: Higher O_2_Hb increase during pre-task (compared to elderly). Both groups: lower activation during pre-task is associated with higher O_2_Hb change during working memory task.
**^∗^ Dupuy** (Front Hum Neurosci., 2015), Canada (Dupuy et al., 2015)	*N* = 58 Healthy older adults (62.9 ± 5.4); young adults (24.6 ± 3.6). Prefrontal.	Task: –Modified Stroop-task with two conditions: naming (identify the color of the ink); executive or incongruent (color of the ink not matching the color-word displayed). Four trial-blocks (60 s each). Rest: 60 s between blocks.	⇑: High-fit women showed increased activation in right inferior frontal gyrus (independent of age group).
**^∗^ Hyodo** (Neuroimage, 2015), Japan (Hyodo et al., 2016)	*N* = 60 Healthy older adults (70.3 ± 3.2). Prefrontal.	Task: –Modified Stroop-task: participants asked to decide if word is printed in the color written below the word (neutral and incongruent conditions). 60 trials (30 neutral and 30 incongruent trials). Rest: 9 –13 s interstimulus interval.	Higher fitness levels and left-lateralized PFC activation related to shorter Stroop interference time. Higher fitness associated with more left-lateralized activation.
**Laguë-Beauvais** (Brain and Cognition, 2015), Canada (Laguë-Beauvais et al., 2015)	*N* = 35 Healthy older adults (63.47 ± 3.67); young adults (23.94 ± 2.32). Prefrontal.	Task: –Color task: identify color of an “X” on screen (by typing on keyboard). –Letter task: identify “K” or “L” on screen (by typing on keyboard). ∙ Conditions: –Single pure: only an “X” or letter is displayed to perform one of the tasks. –Dual mixed: both an “X” and a letter are displayed and have to be answered. These are performed under the instruction to prioritize the letter over the color task (Priority Block) or to give the same priority to both tasks (Equal Block). Rest: staring at fixation cross on screen (1000 ms).	Priority condition: –Older adults: activation in the left dorsolateral prefrontal cortex and bilateral ventrolateral cortex during DT. –Young adults: dual mixed trials showed greater changes in more frontal areas, especially right sided. Equal condition: –Older adults: dual mixed trials engaged bilateral dorsolateral prefrontal cortex, compared to single trials. –Young adults: no differences between activation during dual mixed and single trials. Single trials showed change in activation in right posterior dorsolateral prefrontal cortex for HHb. The activation change between priority and equal conditions was found only in older adults.
**Bierre** (J Gerontol A Biol Sci Med Sci., 2017), New Zealand (Bierre et al., 2017)	*N* = 72 Healthy older adults (66 ± 3.8); young adults (21.9 ± 2.7). Frontal.	Task: –Visuomotor tasks (increasing executive demand): 1)Basic visuomotor performance. 2)Adding inhibition. 3)Adding need to switch between tasks. Rest: 2 min (sitting).	⇑: Older adults showed increased O_2_Hb in relation to increasing task difficulty. Older adults showed higher O_2_Hb compared to younger adults
**Halliday** (Neurophoton., 2017), Canada (Halliday et al., 2017)	*N* = 25 Healthy older adults (75.88 ± 3.28) Prefrontal.	Task: –Computerized cognitive task: Multi-Source Interference Task (congruent and incongruent condition). Fifteen trials in a 30 s block. Rest: 60 s baseline before task. 20 s between blocks.	Greater mean O_2_Hb during congruent (easier) task associated with faster performance and during incongruent (more difficult) task, with slower performance. Greater O_2_Hb variability at within-person level associated with better accuracy and faster performance. Greater O_2_Hb variability at between-person level associated with slower performance.
**Huppert** (PLoS One, 2017), USA (Huppert et al., 2017)	*N* = 19 Older adults (88.1 ± 6.0). Frontal.	Tasks: –Stroop Test. –Symbol Digit Coding. –Shifting Attention Test. Rest: 30 s (quiet sitting baseline).	⇑: Left Broadmann’s area (BA) 10 (right superior frontal) activation during Symbol Digit Coding and Shifting Attention Test. Right BA-10, right BA-45 and left BA-10 activated during Stroop test.
^∗^ **Halliday** (J Clin Exp Neuropsychol., 2018), Canada (Halliday et al., 2018)	*N* = 27 Older adults (76.1 ± 3.3). Prefrontal.	Task: –Computerized cognitive task: Multi-Source Interference Task (congruent and incongruent condition). Fifteen trials in a 30 s block (total of 4 blocks for each condition). Rest: No rest time specified.	⇑: Fallers: activation during congruent and incongruent task; recruited additional tissue to perform at similar level. Non-fallers: no active channels during congruent task; little activation during incongruent task (medial right prefrontal cortex).
Memory test			
**Uemura** (Int J Geriatr Psychiatry, 2016), Japan (Uemura et al., 2016)	*N* = 130 Amnestic MCI (71.8 ± 43); healthy older adults (71.7 ± 3.9). Prefrontal.	Task: –Encoding and retrieval of 10 words (20–30 s respectively). Repeat vowels: Pre-task (10 s), rest after task (20–30 s) and post-task (10 s).	⇓: Reduced activation in bilateral dorsolateral cortex during memory retrieval in amnestic MCI. No significant group effects during encoding.

*AD, Alzheimer’s Disease; DT, dual-task; fNIRS, Functional Near-Infrared Spectroscopy; HHb, deoxygenated hemoglobin; MCI, Mild Cognitive Impairment; O_2_Hb, oxygenated hemoglobin; PFC, Prefrontal Cortex; ST, single task; SD, Standard Deviation; VF, Verbal Fluency. ^∗^Articles assessing modulation of health characteristics on brain activation (Table 3).*

**TABLE 2:** Summary of the studies assessing fNIRS measures during motor and/or dual-tasks in older adults.

First author (Journal, year), Country	Sample size (N) Clinical characteristics of the sample (mean age +SD) NIRS optodes localization	Paradigm description	Main fNIRS results
**(2A) Motor tasks**			
**Mahoney** (Brain Res., 2016), USA (Mahoney et al., 2016)	*N* = 269 Parkinsonian syndrome (81.2 ± 5.9); Mild parkinsonian signs (77.5 ± 6.7); healthy adults (74.4 ± 6.1). Prefrontal.	Task: –Postural control while standing and silently counting for 10 sec.	⇑: Parkinsonian syndromes: increased prefrontal activation to maintain postural control (compared to the other two groups).
**Maidan** (Brain Topogr., 2017), Israel (Maidan et al., 2017)	*N* = 49 PD without cognitive impairment or freezing of gait (72.8 ± 1). Frontal.	Task: –Walk with turns: 30-m walk and 180° turn. –Five trials: 20 s of quiet standing between walk and turn. Rest: 20 s before and after each walk (quiet standing).	⇑: Increased activation during walking and decrease during turns (compared to baseline). ⇑: Older adults with lower gait speed (<1 m/sec): higher activation during turns (compared to older adults with normal gait speed).
**Thumm** (Gait Posture, 2018), Israel (Thumm et al., 2018)	*N* = 20 PD (69.8 ± 6.4). Prefrontal.	Task: –30-m over-ground vs. treadmill walking. –Five trials (30 s each). Rest: 20 s quiet standing.	⇓: Lower activation during treadmill walking (compared to over-ground walking).
**(2B) Dual-tasks**			
**Doi** (Aging Clin Exp Res., 2013), Japan (Doi et al., 2013)	*N* = 16 Older adults with MCI (75.4 ± 7.2). Prefrontal.	Tasks: –ST: 10-m walk. –DT: 10-m walk + phonetic verbal fluency. –Three trials in each condition (20 s each). Rest: 10 s pre-task and 30 s post-task (standing).	⇑: Increased prefrontal activation during DT walking compared to ST walking.
**Ohsugi** (BMC Neurosc., 2013), Japan (Ohsugi et al., 2013)	*N* = 35 Healthy older adults (77.9 ± 5.3) vs. young (26 ± 3.6). Prefrontal.	Tasks: –ST: seated stepping while forward counting from 0. –ST: serial 7-subtraction from 100. –DT: stepping + subtraction. –Each task repeated three times (30 s each). Rest: 30 s (self-paced counting).	⇑: Higher O_2_Hb values during DT compared to stepping as ST. ST count showed higher activation compared to stepping. ⇑: Older adults: higher O_2_Hb levels during DT compared to younger adults.
**Beurskens** (Int J Physchophysiol., 2014), Germany (Beurskens et al., 2014)	*N* = 25 Healthy older adults (71.0 ± 3.8) vs. younger adults (24.5 ± 3.3). Prefrontal.	Tasks: –ST: treadmill walk. –ST: checking boxes on paper. –ST: reciting alternate alphabet. –DT: walk + check. –DT: walk + alphabet. Each task: 30 s and repeated twice. Rest: seated (duration is not specified).	⇓: Older adults: lower activation during walk + check compared to ST walk. No significant difference between walk + alphabet vs. walk. Young: no significant difference in activation during ST vs. DT. ⇑: Higher activation in younger adults compared to older adults during visually demanding dual-task (walk + check).
**Clark** (Front Aging Neurosci., 2015) (Clark et al., 2014)	*N* = 16 Older adults with mild mobility difficulties (77.2 ± 5.6). Prefrontal.	Tasks: –ST: 90-meter walk (5 × 18 m). –Walk + phonetic verbal fluency. –Walk + dimmed light. –Walk + carrying tray. –Walk + 6 obstacles negotiation. –Walk + weighted vest. Rest: 1 min quite standing between tasks.	⇑: Increased activation during DT walk + verbal fluency, walk + vest, walk + obstacles. Although not significative, there was a trend toward increase during DT walk carrying tray and walk with dimmed light.
**Holtzer** (Neuroimage, 2015), USA (Holtzer et al., 2015)	*N* = 348 Healthy older adults (76.8 ± 6.8). Prefrontal.	Tasks: –ST: Walk 3 loops on 14-feet walkway. –ST: 30 s reciting alternate alphabet. –DT: Walk + alphabet. Rest: 10 s standing still and counting silently before tasks.	⇑: Bilateral increases in O_2_Hb during DT compared to normal walk. In ST walk, after an initial increase, O_2_Hb levels decrease in the course of the walk. While during the DT walk, O_2_Hb remains elevated during the task.
**Al-Yahya** (Neurorehabil Neural Repair, 2016), UK (Al-Yahya et al., 2016)	*N* = 19 Chronic stroke (66.2 ± 8.3); healthy controls (56.2 ± 9.5). Prefrontal.	Tasks: –ST: feet tapping. –ST: backward count. –DT: feet tap + count. Five trials (30 s for each task). Rest: 25–45 s in a pseudo-random order after each task.	⇑: Higher O_2_Hb during DT compared to ST in stroke participants compared to healthy controls.
**Hernández** (J Neurol Sci., 2016), USA (Hernandez et al., 2016)	*N* = 16 Multiple Sclerosis (57 ± 5); healthy controls (61 ± 4). Prefrontal.	Tasks: –ST: Walk 3 loops on walkway. –ST: 30 s reciting alternate alphabet. –DT: Walk + alphabet. Rest: 10 s standing still and counting silently before tasks.	⇑: Higher O_2_Hb levels in MS compared to healthy controls in walking tasks. Larger increase in O_2_Hb from ST walk to DT in MS compared to healthy controls.
^∗^ **Holtzer** (Brain Topogr, 2016), USA (Holtzer et al., 2016)	*N* = 236 Healthy older adults (75.5 ± 6.5). Prefrontal.	Tasks: –ST: Walk 3 loops on 14-feet electronic walkway. –ST: Reciting alternate alphabet (30 s). –DT: Walk + alphabet. Rest: 10 s standing still and counting silently before tasks.	⇑: Normal gait: Higher O_2_Hb levels in DT compared to ST walk. ⇓: Central NGA: attenuated changes in PFC O_2_Hb levels from ST to DT compared to peripheral NGA and normal gait group. ⇑: Peripheral NGA showed greatest increase in O_2_Hb during DT.
**Maidan** (Neurorehabil Neural Repair, 2016) (Maidan et al., 2016)	*N* = 106 PD (71.6 ± 0.9); healthy older adults (70.4 ± 0.9). Prefrontal.	Tasks: –ST: Walk on 30-m walkway (30 s). –DT: Walk + serial subtractions. –DT: Walk + obstacles. 5 trials each task. Rest: 1 min before whole paradigm starts and 20 s standing before and after tasks.	⇑: Increased frontal activation during DT walking compared with ST walking in healthy group. ⇓: In PD, HbO_2_ levels did not increase during DT. ⇑: In PD, HbO_2_ increased during walk + obstacle compared with ST walking. ⇑: Higher increase in activation during ST walking in PD compared to healthy controls. No significant difference between groups during DT walks.
**Nieuwhof** (Pilot Feasibility Stud., 2016), Netherlands (Nieuwhof et al., 2016)	*N* = 12 Parkinson’s Disease (70.1 ± 5.4). Prefrontal.	Tasks: –DT: Walk + counting forward. –DT: Walk + serial 3 or 7-substraction. –DT: Walk + reciting digit spans. Five blocks (with 3 tasks each); 40 s each task. Rest: 20 s still-standing before/after task and 1–2 min random rest (while listening to instructions). At least 1 min stand before block.	⇑ All tasks increased O_2_Hb during task compared to rest.
**^∗^ Osofundiya** (Clin Biomech., 2016), USA (Osofundiya et al., 2016)	*N* = 20 Cognitively healthy older adults: obese (80.5 ± 6.8) vs. non-obese (80.6 ± 7.5). Prefrontal	Tasks: –ST: walk (30 s). –DT: walk + reciting alphabet (30 s). –DT: walk and step on targets on walkway (precision walk). Two blocks: 4 trials (30 s each) per block. Rest: quiet sitting (30 s) before start; 10 s quiet standing between trials; 2 min seating between blocks.	⇑: Higher PFC activation during DT and precision walk compared to ST walk. ⇑: Obesity associated greater activation in all tasks but specially during precision walking.
**Takeuchi** (BMC Neurosci., 2016), Japan (Takeuchi et al., 2016)	*N* = 31 Healthy older adults (71.7 ± 3.3); young adults (25.9 ± 4.4) Prefrontal.	Task: –ST: walk for 30 s around a 2.5 m-radius circle –ST: smartphone-based touch game (sitting). Participants instructed to touch in ascending order a set of numbers on screen. –DT: walk + touch. Rest: not described.	No difference between young vs. old in PFC activation during DT. Less PFC lateralization in older adults to suppress DT cost in gait performance.
**Chaparro** (J Neuroeng Rehabil., 2017), USA (Chaparro et al., 2017)	*N* = 22 Healthy older adults (63.1 ± 4.4); multiple sclerosis (56.2 ± 5.1) Prefrontal.	Task: –ST: alternate alphabet reciting (standing) –ST: Normal walk –DT: walk while reciting alternate alphabet Walk: 30 s warm-up walk; 30 s test; 15 s deceleration Rest: 10 s before each task (quiet standing). Protocol performed with and without partial body weight support	⇑: Higher activation during DT compared to normal walk. ⇑: MS older adults: larger increase in O_2_Hb during all tasks in all conditions compared to healthy older adults (especially during DT without partial body weight support).
**Chen** (Gait Posture, 2017), USA (Chen et al., 2017)	*N* = 90 Healthy older adults (78 ± 15.5). Prefrontal.	Tasks: –ST: Walk 3 loops on 14ft electronic walkway. –DT: Walk + alternate alphabet reciting. –ST: Walk with obstacle negotiation. –DT: Walk with obstacle + alternate alphabet reciting. Rest: 10 s standing still and counting silently before tasks.	⇑: Higher activation during DT compared to ST in both normal walk and walk with obstacles. ⇑: Participants with slower gait showed higher increase in O_2_Hb during walk with obstacles compared to unobstructed walk (relative to participants with normal gait).
^∗^ **Holtzer** (Eur J Neurosci., 2017), USA (Holtzer et al., 2017a)	*N* = 318 Healthy older adults (76.6 ± 6.7). Prefrontal.	Tasks: –ST: Walk (3 loops on 14-feet electronic walkway). –ST: Reciting alternate alphabet (30 s). –DT: Walk + alphabet. Rest: 10 s standing still and counting silently before tasks.	⇓: Higher levels of perceived task-related stress associated attenuation of brain activation from ST to DT.
**^∗^ Holtzer** (J Gerontol A Biol Sci Med Sci., 2017), USA (Holtzer et al., 2017b)	*N* = 314 Healthy older adults (76.8 ± 6.7). Prefrontal.	Tasks: –ST: Walk 3 loops on 14-feet electronic walkway. –ST: Reciting alternate alphabet (30 s). –DT: Walk + alphabet. Rest: 10 s standing still and counting silently before tasks.	⇑: Increased O_2_Hb levels during DT walking compared with ST walking. ⇓: Higher levels of subjective fatigue attenuated the increase in O_2_Hb from ST to DT walking.
**Mirelman** (Brain Cogn., 2017), Israel (Mirelman et al., 2017)	*N* = 43. Healthy older adults (69.7 ± 5.8); younger adults (30.9 ± 3.7). Prefrontal.	Tasks: –ST: Walk on 30-m walkway. –DT: Walk + serial subtraction. –DT: Walk + obstacles. Three loops on walkway for 30 s for each task. Rest: 20 s quiet standing before/after tasks.	⇑: Older participants increased O_2_Hb during DT compared to ST walk and during ST walk compared to rest periods. ⇑: Young adults: Activation during DT compared to ST walk. No increase in O_2_Hb during ST walking (compared to rest). ⇑: Older adults showed higher O_2_Hb levels in all tasks compared to younger participant.
**Rosso** (Gait Posture, 2017), USA (Rosso et al., 2017)	*N* = 16 Healthy older adults (74 ± 5); younger adults (24 ± 3). Left prefrontal, temporal, and motor.	Tasks: –ST: Attention task (seated). –ST: Postural control (standing). –DT: Postural control + attention task. Three trials of each task (121 s each). Rest: 30 s sitting or standing before and after each task.	⇑: Older adults had greater activation of prefrontal and temporal regions compared to younger adults.
^∗^ **Verghese** (Neurology, 2017), USA (Verghese et al., 2017)	*N* = 166 Healthy older adults (74.9 ± 6.1). Prefrontal.	Tasks: –ST: Walk 3 loops on 14-feet walkway. –ST: Reciting alternate alphabet (30 s). –DT: Walk + alphabet. Rest: 10 s standing still and counting silently before tasks.	⇑: DT walk showed higher PFC activation than ST walk. Higher PFC activation levels on fNIRS during DT predicted incident falls.
**Mori** (Gait Posture, 2018), Japan (Mori et al., 2018)	*N* = 28 Post-stroke (>6 months) with hemiparesis (61.1 ± 9.3); healthy controls (66.3 ± 13.3). Prefrontal.	Tasks: –ST: Serial subtractions of 3 (standing). –DT: Walk around a circle with 2.5 m radius + serial subtractions of 3. 3 trials. Rest: 60 s (repeat sequence of numbers 1–10).	⇓: Stroke participants: Lower PFC activation during DT compared to healthy participants.
**Hawkins** (Hum Mov Sci., 2018), USA (Hawkins et al., 2018)	*N* = 48 Post-stroke (>4 years) with hemiparesis (58.0 ± 9.3); older adults with mild gait deficits (77.2 ± 5.6); young healthy adults (22.4 ± 3.2). Prefrontal.	Tasks: –ST: Walk on an 18-m oval-shaped course. –DT: Walk + obstacle negotiation. –DT: Walk + phonetic verbal fluency. Rest: quiet standing (duration not specified).	⇑: Elderly vs. young: Higher O_2_Hb increase during normal walk and obstacle negotiation in the early time period. In the late time period, higher PFC activation during normal walk (but not with obstacles). ⇑: Stroke participants: Higher O_2_Hb increase during normal walk and obstacle negotiation compared to young participants. Greater activation during obstacle negotiation compared to elderly in the late time period. O_2_Hb increase was highest in the post-stroke group, followed by older and young adults.
^∗^ **Holtzer** (Brain Cogn., 2018), USA (Holtzer et al., 2018a)	*N* = 315 Healthy old (76.8 ± 6.7). Prefrontal.	Tasks: –ST: Walk 3 loops on 14-feet walkway. –ST: Reciting alternate alphabet (30 s). –DT: Walk + alphabet. Rest: 10 s standing still and counting silently before tasks.	⇑: Participants without diabetes: increased O_2_Hb levels during DT compared to ST walk. ⇓: Diabetes: attenuated increase in O_2_Hb levels from ST walk to DT (compared to non-diabetics).
^∗^ **Lucas** (J Gerontol A Biol Sci Med Sci., 2018), USA (Lucas et al., 2018)	*N* = 55 Healthy older adults (74.7 ± 4.9). Prefrontal.	Tasks: –ST: Walk 3 loops on 20-feet walkway. –DT: Walk + reciting alternate alphabet. Rest: 10 s standing still and counting silently before tasks.	⇑: Higher PFC activation during DT compared to ST. ⇑: Poorer white matter integrity associates greater increase in O_2_Hb levels during DT.

*AD, Alzheimer’s disease; DT, dual-task; fNIRS, Functional Near-Infrared Spectroscopy; MCI, Mild Cognitive Impairment; NGA, Neurological Gait Abnormalities; O_2_Hb, oxygenated hemoglobin; PD, Parkinson’s disease; PFC, Prefrontal Cortex; ST, single task; SD, Standard Deviation; VF, Verbal Fluency. ^∗^Articles assessing modulation of health characteristics on brain activation (Table 3).*

Some studies, beyond assessing frontal hemodynamics, investigated the influence of other clinical characteristics in the reported frontal activation findings (Albinet et al., 2014; Dupuy et al., 2015; Hyodo et al., 2016; Osofundiya et al., 2016; Holtzer et al., 2016, 2017a,b, 2018a; Verghese et al., 2017; Halliday et al., 2018; Lucas et al., 2018) (see Table 3).

**TABLE 3:** Studies assessing effect modification by different health characteristics on PFC activation.

**First author (Journal, year) (reference)**	**Clinical variables**
Halliday et al. (J Clin Exp Neuropsychol., 2017) (Halliday et al., 2018)	Fallers versus non-fallers
Holtzer et al. (Brain Topogr, 2016) (Holtzer et al., 2016)	Neurological Gait Abnormalities
Osofundiya (Clin Biomech., 2016) (Osofundiya et al., 2016)	Obesity
Holtzer et al. (Eur J Neurosci, 2017) (Holtzer et al., 2017a)	Levels of perceived task-related stress
Holtzer et al. (J Gerontol A Biol Sci Med Sci., 2017) (Holtzer et al., 2017b)	Fatigue
Verghese et al. (Neurology, 2017) (Verghese et al., 2017)	Longitudinal association with falls
Holtzer et al. (Brain Cogn., 2018) (Holtzer et al., 2018a)	Diabetes
Lucas et al. (J Gerontol A Biol Sci Med Sci., 2018) (Lucas et al., 2018)	Relation with white matter integrity
Hyodo et al. (Neuroimage, 2015) (Hyodo et al., 2016)	Fitness levels
Albinet (Front Aging Neurosci., 2014) (Albinet et al., 2014)	
Dupuy (Front Hum Neurosci., 2015) (Dupuy et al., 2015)	

### Studies Assessing the Effect of Cognitive Tasks

We found 20 articles assessing cerebral activation during cognitive tasks (Table 1). The most frequent cognitive task was VF (Heinzel et al., 2013, 2015; Yeung et al., 2016a; Yap et al., 2017; Katzorke et al., 2018). Generally, VF tests ask the participants to produce the maximum number of words starting with a specific letter (phonemic) or belonging to a pre-specified semantic category (semantic). Three studies used N-back tests (Niu et al., 2013; Vermeij et al., 2014; Yeung et al., 2016b), which assess working memory function. N-back tasks are usually designed as conditions with increasing working-memory load: 0-back (subject has to detect if the presented stimulus is the one described as target), 1-back (the subject has to remember if the presented stimulus was presented on the previous position) and 2-back conditions (the participant must be able to remember if the stimulus is the same presented 2 positions before). From the twelve remaining studies, eleven used different tests of executive functions (i.e., Stroop, symbol digit coding and shifting attention test, Go/No go inhibition task, Trail Making Test part B, etc.) (Heilbronner and Münte, 2013; Albinet et al., 2014; Müller et al., 2014; Oboshi et al., 2014; Dupuy et al., 2015; Laguë-Beauvais et al., 2015; Hyodo et al., 2016; Bierre et al., 2017; Huppert et al., 2017; Halliday et al., 2017, 2018) and one used an episodic memory task (Uemura et al., 2016).

#### Cognitively Healthy Older Adults

Regarding the studies that assessed frontal hemodynamics in cognitively healthy older adults, two studies by Heinzel et al. (2015) showed different activation patterns while performing VF tasks: one showed an increased activation and another found a decreased activation on bilateral inferior frontal junction in healthy older adults while middle frontal gyri and supramarginal gyri showed an increased activation (interpreted as compensatory mechanisms) (Heinzel et al., 2013). Cognitively healthy older adults showed an increased prefrontal activation while performing a working memory task with visual recognition (Oboshi et al., 2014) as well as with increasing working memory load during a N-back task (Vermeij et al., 2014). Studies using other executive function tests, found an increase in frontal lobe activation during executive function tasks (Heilbronner and Münte, 2013; Albinet et al., 2014; Müller et al., 2014; Bierre et al., 2017; Huppert et al., 2017). One study, instead of reporting only the mean values of O_2_Hb, addressed the association between O_2_Hb variability and behavioral results during an executive function task (Halliday et al., 2017). They reported that within-person O_2_Hb variability was associated with better accuracy and faster performance but between-person variability was associated with slower performance.

#### Comparison of Healthy Old Versus Young Adults

Healthy older adults showed higher frontal activation than younger persons while performing a visuomotor task with increasing executive function demand (Bierre et al., 2017). Moreover, a different activation pattern during executive function tests between age groups was observed. According to Heilbronner and Münte (2013), in older adults, activation shifted rostrally on the left hemisphere and dorsally on the right hemisphere during the inhibition task, while Müller et al. (2014) reported an additional activation in left medial and lateral PFC during the TMT-B (while more ventral activation was evidenced in younger counterparts). The effect of prioritization of a stimulus was assessed in one study (Laguë-Beauvais et al., 2015) where the participants were asked to prioritize one of two stimuli displayed (priority block) or to give the same priority to both stimuli (equal block). A change in the activation pattern between the priority and equal conditions was found only in the older adults group, with a less lateralized pattern (bilateral dorsolateral PFC activation) when not prioritizing either stimuli.

#### Comparison by Cognitive Status

Regarding the studies assessing older adults with different cognitive status, three studies using VF tasks reported an increased activation during the task in MCI (Yeung et al., 2016a; Yap et al., 2017) and mild AD (Yap et al., 2017) while Katzorke et al. (2018) found a decreased activation during VF in MCI patients. Yap et al. (2017) compared the activation pattern in cognitively healthy older adults, MCI and mild AD and found the highest O_2_Hb increase in MCI older adults followed by healthy and AD participants, although the difference was not statistically significant. Increasing working memory load led to lower frontal lobe activation during a N-back task in MCI, compared to healthy controls (Niu et al., 2013; Yeung et al., 2016b). Only one study measured PFC activation during encoding and retrieval of episodic memory, and it found a decreased activation on bilateral dorsolateral cortex during memory retrieval in amnestic MCI (Uemura et al., 2016).

### Studies Assessing the Effect of Motor Tasks

All the studies that used isolated motor tasks in order to assess PFC hemodynamics (*n* = 3) enrolled older adults with parkinsonian syndromes but were heterogeneous regarding the motor tasks paradigm (Table 2A). The reported results were also heterogeneous. According to Mahoney et al. (2016), older adults with parkinsonian syndromes showed higher PFC activation while performing a postural control task (compared to participants with mild parkinsonian signs or without these). Participants with PD walking on a straight walkway showed an increased PFC activation, compared to the baseline, and a decrease when performing 180° turns (Maidan et al., 2017). However, when comparing older adults with different gait speed, participants with gait speed lower than 1m/sec showed higher activation during turns, compared to those with normal gait speed. Thumm et al. reported lower O_2_Hb levels while walking on a treadmill vs. over-ground walking in PD participants (Thumm et al., 2018).

### Studies Assessing the Effect of Dual Tasking

Twenty-three articles assessed PFC hemodynamics while performing DT (Table 2B). Studies included in this review used walking (Doi et al., 2013; Beurskens et al., 2014; Clark et al., 2014; Holtzer et al., 2015, 2016, 2017a,b, 2018a, 2018b; Maidan et al., 2016; Nieuwhof et al., 2016; Takeuchi et al., 2016; Chaparro et al., 2017; Chen et al., 2017; Mirelman et al., 2017; Verghese et al., 2017; Hawkins et al., 2018; Lucas et al., 2018; Mori et al., 2018), feet tapping (Al-Yahya et al., 2016), stepping (Ohsugi et al., 2013) and postural control (Rosso et al., 2017) as the motor task and VF (Doi et al., 2013; Clark et al., 2014; Hawkins et al., 2018), calculation (Ohsugi et al., 2013; Al-Yahya et al., 2016; Maidan et al., 2016; Nieuwhof et al., 2016; Mirelman et al., 2017; Mori et al., 2018), alphabet (Beurskens et al., 2014; Holtzer et al., 2015, 2016, 2017a,b, 2018; Chaparro et al., 2017; Chen et al., 2017; Verghese et al., 2017; Lucas et al., 2018), digit span (Nieuwhof et al., 2016), visual (Beurskens et al., 2014) or attention (Takeuchi et al., 2016; Rosso et al., 2017) tasks as the added cognitive tasks. Other studies used challenging factors while walking such as obstacle negotiation or carrying a tray as the secondary task to assess DT performance (Clark et al., 2014; Maidan et al., 2016; Osofundiya et al., 2016; Chen et al., 2017; Mirelman et al., 2017; Hawkins et al., 2018).

#### Cognitively Healthy Older Adults

The vast majority of studies reported an increase in PFC activation in cognitively healthy older adults while performing several types of DT compared to a ST (Ohsugi et al., 2013; Clark et al., 2014; Holtzer et al., 2015, 2017a,b; Maidan et al., 2016; Osofundiya et al., 2016; Chen et al., 2017; Mirelman et al., 2017; Verghese et al., 2017; Lucas et al., 2018). Only one article reported lower O_2_Hb levels during walking while performing a visual check task compared to ST walk in the older adults group (Beurskens et al., 2014).

#### Comparison of Cognitively Healthy Older Versus Younger Adults

Older older adults showed higher PFC activation during DT in most studies, compared to younger participants (Ohsugi et al., 2013; Mirelman et al., 2017; Rosso et al., 2017; Hawkins et al., 2018). Only one study reported lower activation in older adults, compared to younger older adults, during a walk and visual check DT (Beurskens et al., 2014) and Takeuchi et al. (2016) did not find significant differences between age groups.

#### Other Clinical Conditions

The effect of dual tasking in older adults with MCI was assessed in one of the included studies, which found an increased activation during DT compared to ST walking (Doi et al., 2013). Frontal hemodynamics has also been studied in stroke patients, although these studies included participants with heterogeneous clinical characteristics (mainly the time after the stroke event) and DT paradigms (i.e., Task protocols). Compared to healthy controls, patients with stroke history showed higher activation during counting while feet tapping (Al-Yahya et al., 2016) but a lower activation during counting while walking in another study (Mori et al., 2018). Walking while negotiating obstacles caused a higher activation in stroke patients compared to younger adults (Hawkins et al., 2018). PD patients show an increase in frontal activation during DT that involve walking and counting or reciting digit spans compared to the resting baseline periods (Nieuwhof et al., 2016). Middle-aged Multiple Sclerosis older adults show increased PFC activation during ST and DT walking and larger increases in O_2_Hb levels from ST to DT when compared to healthy older adults (Hernandez et al., 2016; Chaparro et al., 2017). Multiple Sclerosis participants show an especially larger increase in activation (compared to healthy counterparts) when not provided with partial body weight support (Chaparro et al., 2017).

### Association Between Activation and Clinical Variables

Other studies assessed how different variables modulate the PFC activation during cognitive, motor tasks and DT (Table 3). Publications from the “Central Control of Mobility in Aging” (CCMA) study, including community-dwelling older adults without dementia, found that activation of PFC during DT, compared to ST, was lower in participants with central NGA compared to peripheral NGA or with normal gait. In fact, the highest O_2_Hb increase during DT was showed by participants with peripheral NGA (Holtzer et al., 2016). Also in participants from the CCMA study, higher levels of self-perceived stress and fatigue were associated with attenuation of brain activation patterns (lower increase in O_2_Hb levels from ST to DT walking) (Holtzer et al., 2017a, b). Participants with diabetes from the same study showed lower PFC activation during DT, compared to non-diabetics (Holtzer et al., 2018a), while obese cognitively healthy older adults from a different study showed higher activation, especially during a precision walking task, compared to non-obese counterparts (Osofundiya et al., 2016). When combining fNIRS with cerebral microstructural white matter integrity assessment, using MRI with Diffusion Tensor Imaging (DTI), altered white matter integrity was associated to higher O_2_Hb levels during DT walk compared to normal walk in the CCMA study (Lucas et al., 2018). Using data from the same study, Verghese et al. (2017) revealed higher risk of incident falls in older adults with higher levels of PFC activation during DT. It is important to note that this is the only article included in our review that assessed the relationship between PFC hemodynamic and outcomes in a longitudinal manner. Furthermore, in a separate sample, fallers compared to non-fallers (history of falls in the previous 2 years) had higher activation while performing executive function tasks (Halliday et al., 2018). The effect of fitness level on frontal activation during executive functions tasks among cognitively healthy participants was addressed in three studies. Although they assessed the level of fitness with different instruments, it seems that higher levels of fitness might produce larger increases in prefrontal activation (Albinet et al., 2014). Two of these studies used two different versions of modified Stroop tasks and while one found a more left-lateralized activation in the high-fit participants (Hyodo et al., 2016), the other study found an increased activation in right inferior frontal gyrus in the high-fit group (Dupuy et al., 2015).

## Discussion

### Summary and Interpretation of Findings

Our review identified 46 articles that reported the assessment of frontal and PFC hemodynamics in older adults using fNIRS during cognitive, motor and DTs.

This has revealed a quite homogeneous pattern of activation of the PFC in cognitively healthy older adults during cognitive and DTs compared to rest and to single-task conditions, respectively. This supports the use of fNIRS investigations to detect changes in frontal hemodynamics in older adults.

Cognitively healthy older adults, compared to younger ones, show a higher activation during executive function tasks and DTs (Ohsugi et al., 2013; Bierre et al., 2017; Mirelman et al., 2017; Rosso et al., 2017; Hawkins et al., 2018). However, one study reported lower activation during walking while performing a visual check task compared to ST walk in the older adults group and compared to the younger group (Beurskens et al., 2014). The results in older adults with various degrees of cognitive impairment are more heterogeneous. Overall, MCI older adults show increased PFC activation during VF tasks (Yeung et al., 2016a; Yap et al., 2017) and during DT compared to ST (Doi et al., 2013). However, gradually increasing working memory load causes a lower activation compared to healthy controls (Niu et al., 2013; Yeung et al., 2016b).

These findings are in line with previously proposed hypotheses, such as the “neural inefficiency theory” (Rypma and D’Esposito, 2000; Holtzer et al., 2009), according to which older adults show increased activity of the same networks recruited by younger counterparts in order to meet behavioral demands. On the other hand, the lower activation in the healthy old subgroup relative to younger adults could be interpreted as an inability to meet the increased cognitive demands during the more complex DT (Beurskens et al., 2014) and is supported by the “capacity limitation hypothesis” (Cabeza, 2004; Holtzer et al., 2009). This theory might also explain the decrease in activation in MCI older adults with increasing working memory load (Niu et al., 2013; Yeung et al., 2016b). Importantly, neural inefficiency and capacity limitation theories are not mutually exclusive and likely both play a role in determining activation levels.

Regarding the studies focusing on older adults with other specific diseases, the findings support an activation of PFC during gait as ST (Maidan et al., 2017) and DT (Nieuwhof et al., 2016) in adults with PD (compared to rest periods). The only study that assessed PFC during postural control found a higher activation in participants with parkinsonian syndromes relative to healthier controls (Mahoney et al., 2016). This could be interpreted in the context of the neural inefficiency theory, where adults with impaired postural mechanisms as seen in PD (Baltadjieva et al., 2006; Benítez-Rivero et al., 2013), need a higher PFC activation to maintain postural control. Similar results, of higher activation than healthy controls, were obtained in Multiple Sclerosis participants (Hernandez et al., 2016; Chaparro et al., 2017) whereas stroke patients reported more heterogeneous results. This might be due to different clinical characteristics of the samples and of the DT paradigms (Al-Yahya et al., 2016; Hawkins et al., 2018; Mori et al., 2018).

Studies that investigated the effect of several clinical variables on the PFC activation during DT found a higher activation in participants with peripheral NGA, lower stress and fatigue levels, obesity, non-diabetics and altered white matter integrity in MRI. The only study that assessed prediction of longitudinal outcomes of frontal hemodynamics, found a higher risk of falls associated with higher PFC activation. However, most of these findings come from a single sample. According to the results from three studies, higher levels of fitness might produce larger increases in prefrontal activation during executive functions tasks in healthy older adults (Albinet et al., 2014; Dupuy et al., 2015; Hyodo et al., 2016).

Overall, our findings suggest that fNIRS studies are able to detect changes in frontal and PFC activation in older adults (both cognitively healthy and MCI), especially while performing cognitive and DTs that are believed to engage the frontal areas of the brain. In particular, in both the comparison between older and younger adults, and in people with different neurological conditions, compared to healthier controls, the PFC seems to experience a higher activation, which could be interpreted in the context of proposed neural inefficiency and limited capacity models.

### Methodological Aspects and Limitations

Main limitations of the fNIRS technique arise either due to physical or technological constraints of the setups, due to analysis methods, or due to the nature of the study itself. It is well known that the recorded signal contains information not only from brain activation due to a specific stimulus or task but is also affected by extra-cerebral (skull and scalp perfusion) as well as systemic parameters (heart and respiratory rate, blood pressure, Mayer waves). Nowadays, the fNIRS community has made not only technological improvements but also has developed an abundance of methods to attempt to overcome the abovementioned limitations (Tachtsidis and Scholkmann, 2016). Current instrumentation provides the ability of using multiple source detector pairs that cover a wide range of tissue penetration depth, giving the possibility to record short channel preparation and regress out signal coming from superficial tissue layers when using continuous wave light sources (Yücel et al., 2015). On the other hand, emerging methods that employ pulsed light sources [time-resolved NIRS (TRS)], allow for the possibility to discriminate between intra- and extra cerebral signals (Torricelli et al., 2014). These methods were prohibitively complex but have recently begun to become practical (Pifferi et al., 2016). In this context, to cover a large imaging area, multiple channels can be used in combination with MRI, thus overcoming the lack of anatomical information and allow for localization of the origin of NIRS signal (Okamoto et al., 2004). Another technical limitation, could originate from the differential path length factor (DPF), used in modified Beer-Lambert law (Cope and Delpy, 1988), that could lead in cross-talk between oxygenated and deoxygenated hemoglobin measurements and false calculations (Hoshi, 2007). Regarding the analysis methods of the acquired fNIRS signal, to date, there is no standard method established (Pfeifer et al., 2018). Some of the most common strategies include the use of low-pass filters to remove heart rate or instrumental noise and high pass filters to extract low frequency systemic noise. Signal analysis methods are also heterogeneous in the current literature (Kirilina et al., 2012; Zhang et al., 2016). Furthermore, in functional studies and especially in motor and DT, motion artifacts play an important role, therefore, motion correction processes are widely used, covering a wide range of proposed methods (Wavelet filtering, Kalman filtering, spline interpolation, etc.) (Cooper et al., 2012; Brigadoi et al., 2014). In general, for more accurate results when designing an fNIRS experimental protocol or analysis method, it is crucial to take into account, potential particularities that each studied population might have.

The heterogeneity in task protocols, methodology and small sample sizes in most of the included articles may limit the interpretation of the findings, although the studies with larger samples show promising results in similar directions. The great majority of the reviewed articles measured activation only over frontal areas, avoiding the assessment of possible compensatory activations in distant areas of the brain (Stern, 2005; Holtzer et al., 2009). This may be mainly due to the simplicity of the application over hairless areas and can be overcome with better probe designs.

Furthermore, differences in cerebral activation patterns detected by fNIRS could be actually related to structural alterations, as recently reported in an MRI-fNIRS study (Wagshul et al., 2019) where higher activation in healthy older adults during DT was related to reduced cortical volumes, especially in bilateral superior and rostral-middle frontal cortex. More evidence is needed supporting this concept.

Other gaps and limitations might limit the generalizability of the results produced by the studies published to date. Regarding studies reporting the results of the motor task alone (not as dual task), the studies are limited to older adults with Parkinson syndromes. The samples are also very heterogeneous regarding the mean age ranges and other clinical characteristics. In most of the included studies, inclusion criteria take into account age and cognitive function, but individuals of the samples or within the comparison groups might be heterogeneous regarding aspects which might affect the cerebral neurovascular coupling and metabolism, such as cardiovascular risk load, atherosclerosis, small vessels disease etc.

Our work is not exempt from limitations. In particular, the non-systematic search strategy might lead to possible missing relevant published literature on the topic. However, we consider our pre-defined search strategy sufficiently comprehensive to include the most if not all relevant ones.

### Clinical Implications and Future Directions

Our findings support the potential role of fNIRS in research and clinical practice to study cognition and mobility in aging. As mentioned, fNIRS is a non-invasive technique, which can assess brain regions involved in executive functions, which are key to goal-oriented behaviors and preserved cognitive and motor functions. In particular, fNIRS allows to obtain relevant information regarding neural activation while the person is performing a real motor task in a natural environment, in a relatively inexpensive way. However, further research is needed to confirm those findings and to establish standardized protocols (for tasks protocols and fNIRS data acquisition and processing). Further research should also focus more on cerebral hemodynamic in different neurological diseases and on the influence of systemic conditions (e.g., vascular risk factors such as diabetes and hypertension) on brain activation patterns as assessed with fNIRS. Furthermore, fNIRS-derived brain activation patterns can be utilized as predictors of incident health outcomes including but not limited to dementia.

A recent study demonstrated that within session training resulted in improved DT walking that was coupled with reduced activation in the PFC among healthy older adults suggesting improved neural efficiency due to practice (Holtzer et al., 2018b). Moreover, the presence of fear of falling delayed practice-related improvements in PFC efficiency during DT walking (Holtzer et al., 2019). These findings suggest that fNIRS can be used to quantify neuroplasticity, monitor improvement in PFC efficiency due to practice and detect the effect of clinically relevant variables such as fear of falling on brain function and efficiency during active walking. Hence, it is appropriate to consider the inclusion of fNIRS at least as a secondary outcome measure in clinical trials designed to assess the effect of treatment on brain neuroplasticity and efficiency as well as for the development and monitoring of rehabilitation/training programs.

## Conclusion

In conclusion, our review supports the use of fNIRS as a neuroimaging technique to study changes in the hemodynamic response in the frontal cortex during cognitively demanding tasks and during active walking under single and DT conditions in older adults. From a pathophysiological perspective this approach might help characterize the evolution of functional impairments in different neurological diseases in older adults as well as in healthy aging.

## Author Contributions

CU and MI designed the concept of this manuscript, reviewed abstracts and full-text publications to assess eligibility, and wrote the first draft of the manuscript. CU extracted the relevant data from the included publications to write the manuscript. SA, TD, RH, AR, CC-T, L-MP, and LS-B contributed in the redaction and revision of the manuscript.

## Conflict of Interest

The authors declare that the research was conducted in the absence of any commercial or financial relationships that could be construed as a potential conflict of interest.

## Abbreviations

AD: Alzheimer’s disease
DT: dual-task
fNIRS: Functional Near-Infrared Spectroscopy
HHb: deoxygenated hemoglobin
MCI: mild cognitive impairment
NGA: Neurological Gait Abnormalities
O_2_Hb: oxygenated hemoglobin
PD: Parkinson’s disease
PFC: prefrontal cortex
ST: single task
VF: verbal fluency.
